# Rationale, design and conduct of a randomised controlled trial evaluating a primary care-based complex intervention to improve the quality of life of heart failure patients: HICMan (Heidelberg Integrated Case Management)

**DOI:** 10.1186/1471-2261-7-25

**Published:** 2007-08-23

**Authors:** Frank Peters-Klimm, Thomas Müller-Tasch, Dieter Schellberg, Jochen Gensichen, Christiane Muth, Wolfgang Herzog, Joachim Szecsenyi

**Affiliations:** 1 Department of General Practice and Health Services Research, University Hospital of Heidelberg, Voßstraße 2, 69115 Heidelberg, Germany; 2 Department of Psychosomatic and General Internal Medicine, University of Heidelberg Hospital, Germany; 3 Institute for General Practice, Chronic Care and Health Services Research University of Frankfurt, Theodor-Stern-Kai 7, 60590 Frankfurt a. M., Germany

## Abstract

**Background:**

Chronic congestive heart failure (CHF) is a complex disease with rising prevalence, compromised quality of life (QoL), unplanned hospital admissions, high mortality and therefore high burden of illness. The delivery of care for these patients has been criticized and new strategies addressing crucial domains of care have been shown to be effective on patients' health outcomes, although these trials were conducted in secondary care or in highly organised Health Maintenance Organisations. It remains unclear whether a comprehensive primary care-based case management for the treating general practitioner (GP) can improve patients' QoL.

**Methods/Design:**

HICMan is a randomised controlled trial with patients as the unit of randomisation. Aim is to evaluate a structured, standardized and comprehensive complex intervention for patients with CHF in a 12-months follow-up trial.

Patients from intervention group receive specific patient leaflets and documentation booklets as well as regular monitoring and screening by a prior trained practice nurse, who gives feedback to the GP upon urgency. Monitoring and screening address aspects of disease-specific self-management, (non)pharmacological adherence and psychosomatic and geriatric comorbidity. GPs are invited to provide a tailored structured counselling 4 times during the trial and receive an additional feedback on pharmacotherapy relevant to prognosis (data of baseline documentation).

Patients from control group receive usual care by their GPs, who were introduced to guideline-oriented management and a tailored health counselling concept.

Main outcome measurement for patients' QoL is the scale physical functioning of the SF-36 health questionnaire in a 12-month follow-up. Secondary outcomes are the disease specific QoL measured by the Kansas City Cardiomyopathy questionnaire (KCCQ), depression and anxiety disorders (PHQ-9, GAD-7), adherence (EHFScBS and SANA), quality of care measured by an adapted version of the Patient Chronic Illness Assessment of Care questionnaire (PACIC) and NT-proBNP. In addition, comprehensive clinical data are collected about health status, comorbidity, medication and health care utilisation.

**Discussion:**

As the targeted patient group is mostly cared for and treated by GPs, a comprehensive primary care-based guideline implementation including somatic, psychosomatic and organisational aspects of the delivery of care (HICMAn) is a promising intervention applying proven strategies for optimal care.

**Trial registration:**

Current Controlled Trials ISRCTN30822978.

## Background

Congestive Heart Failure (CHF) is a disease with high incidence, prevalence, and the cumulative lifetime risk to develop CHF of 20% [1-3]. Despite substantial progress in medical treatment [4], one-year mortality ranges stage-dependently from 7 to 28% and increases to 75% after five years [5,6]. The burden of illness includes multiple acute failures followed by hospital admissions [7]. New treatment strategies focus on preventing readmissions and on improving the prognosis. With a higher stage of CHF the Quality of life (QoL) decreases [8]. Some studies suggest that QoL is a predictor of the course of CHF, independent of the acknowledged somatic predictors of prognosis (like left ventricular ejection fraction) [8,9].

Improvement of mortality rates by pharmacotherapy does not necessarily implicate improvement of QoL[10]. Non-medical treatment like exercise training and patient education have been shown to have a high impact on QoL [11]. However, it has been shown that the known clinical and somatic predictors explain only about 40% of the total variance of QoL [8,12].

Transfer of these new insights has not been implemented in daily practice, although specific care taking psychosocial aspects into account has been demanded [13,14]. However, there are no concepts for specific training dealing with patients with CHF to improve their QoL in Primary Care. Although QoL is a main concern of patients, it is not a central topic of cardiac research.

To date, only small-scale studies with selective samples analysed data regarding QoL in patients with CHF. In addition, there is no horizontal and vertical networking for transfer of knowledge. This is especially the case for the transfer of specific interventions for general practice. There are several guidelines for the management of patients with heart failure[15], but adherence to these guidelines is low [16].

### Novel aspect of integrated case management

To our knowledge, the proposed study is the first investigating the efficacy of a multifaceted case management in patients with CHF in general practice in Germany. Studies showing the efficacy of case management in patients with CHF were performed in highly organised Health Maintenance Organisations and have therefore limited external validity. GPs in Germany as in many other countries work mostly in single or double practices without multi-professional teams. A nurse specialist exerting a form of structured care is new to the German system. The structured care intervention consists of telephone monitoring, home visits with additional diagnostic screening, medication feedback, patient leaflets, and specific counseling allowing assessment of and intervention in a multitude of aspects of care relevant to the patient. Provided that our intervention proves to be effective, the better treatment of patients with CHF in general practice could prevent personal suffering, improve patient and provider satisfaction, and lower health care costs.

### Evidence

The following studies give evidence for the necessity, clinical relevance, and novelty of the trial: Depressive symptoms and quality of life are strongly associated variables[17]. QoL and depressive disorders are predictive for unscheduled readmissions and mortality[8,9,18-20]. In the "Improvement of Heart Failure Initiative Study" the level of knowledge, the diagnostic as well as the therapeutic approach of 100 GPs (900 patients) have been evaluated. As the results show, patients in Germany are treated according to guidelines only in about 20% of the cases. Cardiologists and GPs set different priorities concerning diagnostics and therapy [21,22]. Improvement of mortality rates by pharmacotherapy does not implicate improvement of QoL[10]. Studies evaluating "complex" case management for CHF in or starting from secondary care have shown to have favourable outcome with regard to QoL, readmission and mortality [23]. Sustained effects have been shown in long-term follow-ups [24].

## Methods/Design

### Aims of the study

With the purpose to improve QoL and physical outcomes this study aims to investigate multifaceted case management of patients with CHF in general practice. Therefore, an intervention that specifically addresses the needs of this patient group will be compared to usual treatment as control condition.

#### Scientific hypothesis

The case management intervention group shows a significantly better outcome with respect to QoL compared to the control group at the one-year follow-up. We further expect greater improvements in the intervention subjects compared to controls with respect to readmission and mortality, physical outcomes parameters (NYHA and NT-proBNP), patients' satisfaction with medical care and health service utilization.

### Study/trial design

**Figure 1:**
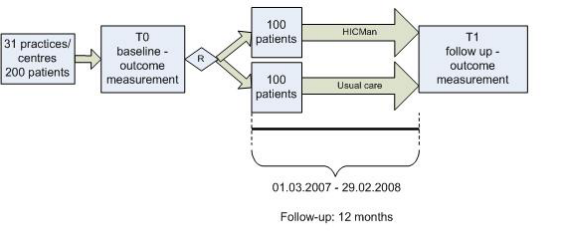
Trial design (HICMan-CHF).

A (prospective,) two-armed, randomised controlled trial will be performed, with the patients as unit of randomisation. This trial design was chosen because of high internal validity, while the number of needed recruited patients remains feasible. Contamination is avoided as the intervention is highly standardized, time-consuming and linked to a personal one-time documentation. Eligible patients will be randomized to either intervention or usual care conditions (see figure 1).

### Sample size/Power calculations

We expect that the differences in QoL between the interventional and control arm to be at least 9% with superior QoL in the interventional arm (physical functioning, Scale 1 of SF-36). With a serial correlation of 0.6, n = 66 patients are needed per arm for 80% power at an alpha-level of 5%. Calculating a drop-out of 30%, 132 cases represent 70%. Therefore, at least 188 patients will be initially included.

### Recruitment of GPs and randomisation

GPs were eligible for randomization if their practice had a contract with all German insurances, ensuring that patients of all social levels have unlimited admission to the practice. About 200 GPs in the area of Baden-Wuerttemberg, fulfilling the inclusion criteria, were invited by a formal letter from the Department of General Practice and Health Services Research of the University Hospital Heidelberg, to participate in the study. 36 GPs gave their written consent to participate in the study. 5 GPs declined to participate prior to randomisation, two due to lack of eligible patients, two due to high time and effort and one because of disagreement concerning the "evidence based" approach. Based on detailed information about the practice and the GP, the inclusion criteria were checked. No GP or no practice had to be excluded due to the inclusion criteria. Of the participating 31 centres 19 took part in the previous train the trainer-study (ttt) about guideline implementation. In this RCT general practitioners (GPs) of the intervention group got a special training in a clinical practice guideline (CPG) on CHF while GPs of the control group merely got the CPG with a standard lecture, and we compared the QoL of CHF patients in both groups. The participating GPs of the ttt-study were invited to continue in the HICMan-study.

From November 2006 to January 2007, 31 recruiting centres (with about 6–7 recruited patients per centre) enrolled 200 eligible patients and obtained informed consent prior to allocation. We used third party fax randomisation, conducted by the Coordination Centre for Clinical Trials Leipzig (KKSL). KKSL obtained the randomisation faxes directly from the centres and conducted the procedure based on a computer generated, concealed random allocation sequence using an algorithm of Pocock: Patients were grouped by the interval of recruitment time. Each group was randomised immediately at the end of the respective recruitment interval, stratified by arm and centre. Assignment of the patients to their intervention arm was sent by fax from KKSL to the centres. The assessors of outcomes are blinded until post-intervention data collection is completed.

### Inclusion/exclusion criteria

#### Case manager inclusion criteria

For the practice nurses who wanted to engage in the study, the following criteria had to be fulfilled apart from the participation in the study's case management work shops: status of practice nurse or at least in the third year of formal training.

#### Patient inclusion criteria

To be eligible for the study, patients had to fulfil the following inclusion criteria: age> = 40 years, objective left ventricular CHF, ejection fraction of 45% or less (confirmed by echocardiography within the last 24 months), no dyspnea (NYHA I), but hospital admission because of CHF within the last 24 months or dyspnea NYHA II-IV, stability of the disease at the time of inclusion, capability to give informed consent.

#### Patient exclusion criteria

To be eligible for the study, the following criteria had to be excluded: participation in another clinical trial or specific care concept within the last 30 days (incl. telemedicine interventions), residency in a nursing home, primary valvular heart disease with relevant hemodynamic effects, Hypertrophic Obstructive/Restrictive Cardiomyopathy (HOCM/RCM), status post/pre organ transplantation, acute left ventricular failure, short life expectancy of < 2 years due to serious concomitant illness, known impaired mental state (e.g. dementia) that prevents accurate answers to questions, addictive disorders with continuing drug abuse despite social, legal or professional conflicts.

### Data collection

After giving their informed and written consent to participate in the study, patients were registered in the Centre of the Competence Network of Heart Failure (Berlin) which is responsible for administration, coordination and pseudonymisation of patients' personal data.

Patients will receive a questionnaire for evaluation of generic QoL (SF-36) and disease-specific QoL (KCCQ) as well as other items to assess secondary outcome parameters as shown in table 1. GPs will document clinical data (history, actual clinical status, lab results, ECG etc.) and draw a blood sample for determination of NT-pro-BNP. Analysis of blood samples will be performed by the Charité Universitätsmedizin Berlin. A study nurse will collect the documents in the practice and forward them to the Coordination Centre for Clinical Trials Leipzig (KKSL, Germany), which is responsible for data management (database set-up and validation, data entry, coding, query management and case report form printing).

**Table 1:** Outcome-parameter and instruments

**Outcome-parameter**	**Instrument**
**Primary outcome**	
Quality of life	SF-36, scale 1 (PF)
	
**Secondary outcomes**	
Quality of life	other scales of SF-36
Disease-specific Quality of life	KCCQ
Depression	PHQ-9
Anxiety disorder	GAD-7
Patient assessment of care	Modified PACIC
Adherence	EHFScBS and SANA
Disease course	NT-proBNP
	
Other comorbidity	CIRS, small geriatric assessment
Health care utilisation	Case report form (CRF)
Medication	CRF
Sociodemographic variables	Questionnaire
**HICMan documentation of CM**	CRF, PHQ-9, GAD-7 and small geriatric assessment

### Outcome-parameter

#### Primary and secondary outcomes

Table 1 displays the outcome parameters and associated instruments used. The primary outcome is quality of life assessed by the Short Form 36 Health Questionnaire (physical function scale)[25], an internationally validated generic instrument for the assessment of quality of life.

Secondary outcomes are the other scales of SF-36[25], disease-specific QoL (KCCQ [26]), signs for depressive and anxiety disorders (PHQ-9 and GAD-7)[27,28], Patient Assessment of Chronic Illness Care (modified PACIC [29]), admission to hospital or death due to heart failure (combined), improvement of heart failure according to functional status (NYHA) and NT-proBNP [30], the European Heart Failure Selfcare Behaviour Scale (EHFScBS[31]), the Scales to Assess Low Adherence (SANA[32]), evidence-based pharmacotherapy and cost-effectiveness. Additionally, the case management will be analysed. This includes the used monitoring list and screening tools (study specific assessment of patients behaviour regarding life style, PHQ-9, GAD-7 and small geriatric assessment [33-35]).

These data will be compiled from patient questionnaires and case report forms of the patients. All instruments except SANA and PACIC represent well established instruments that are validated in Germany. No interim analysis will be performed except for pharmacotherapy to provide prescription feedback.

### Intervention/Follow-ups

Patients randomised to the intervention arm will get case management care by a specifically trained practice nurse (case manager = CM). Duration of training was 1,5 days.

All patients receive an introduction of the CM, monitoring by telephone, home visits, disease-specific counselling, and surveillance concerning prescription. Details are listed below:

1. Introduction: After enrolment, the CM will take approximately 30 min. for introducing her function and establishing a relationship to the patient. Patients get information about their disease, realisation of symptoms and self-monitoring (shortness of breath, fatigue, peripheral oedema, weight, heart rate and blood pressure). On that occasion, patients will get an information booklet and documentation booklet.

2. Monitoring by telephone: The CM carries out a telephone monitoring according to the patients' risk:

• low to medium risk (NYHA I/II) – every six weeks, three personal home visits during the year instead of telephone monitoring.

• high risk (NYHA III/IV) – every three weeks, three personal home visits during the year instead of telephone monitoring.

Content of the telephone interview will check for physical warning physical signs, problems with medication (adherence).

3. Home visits: During home visits (three times a year) certain domains from the following will be evaluated in a structured and operated way tailored to heart failure patients: Physical, cardiac status; structured assessment of lifestyle [36] (as first step part of a 5-A counselling, see below); adherence; depression and anxiety disorder screening (2×); small geriatric assessment (2×) and a detailed medication check (1×).

4. After the introduction and the three home visits, the patients will get a specific physician encounter for counselling as part of a 5-A counselling [37,38] based on the structured assessment of relevant lifestyle and habits in relation to heart failure concerning the topics (i) self management, (ii) physical activity and (iii) smoking.

5. Recall-Reminder-Systems: if necessary, active surveillance concerning prescription or doctor follow-ups will be applied.

6. In month seven, GPs will receive an additional feedback on pharmacotherapy relevant to prognosis for patients from the IG (data of baseline documentation). Substances from the following classes will be transformed from the following three drug classes: ACE-inhibitors (or angiotensin-II receptor antagonists), beta-blockers and aldosterone antagonists. Percentage of reached target dose (from current guideline) will be calculated. The procedure will be performed by a statistician not involved in the conduct of the study. GPs will get a printout showing a graphical depiction of each patient from the IG (he is caring for).

#### Actions to take by the CM

During each visit, the CM will apply a structured and formalised assessment of cardiac/physical functioning and additional screening according to the structured plan, as outlined above. Results of the screening will guide the CM to tangible actions, which consist of a feedback or, if necessary, of direct contact to/with the treating physician upon urgency.

#### Control condition

Participating physicians will receive a guideline for the management of heart failure as the recommended standard and the introduction to a structured counselling for heart failure (5-A). No case management will be applied to the patients of this group.

#### Follow-ups

CM home visits will take place at the beginning, after the first and the second third of the year. Assessment and documentation will take place at the beginning and the end of the year (see figure 1).

### Timeframe of the study

The study has the following phases: Training of study nurse and preparation of trial (months 1 – 6), recruitment (months 1 – 6), treatment/control (months 7 – 18), follow-up (month 18), data entry (months 19–20), data analyses and publication of results (months 21–24). At the time of drafting the manuscript, the planned time schedule is kept: 200 patients were enrolled. The study was in the phase of treatment/control in month 10.

### Description of risks

Serious risks or undesired effects of questionnaires have not been described in the literature. No specific risk for participating patients can be stated. The management of the patients remains in the responsibility of the treating physician.

### Ethical and legal aspects

#### Ethical principles

The study is being conducted in accordance with medical professional codex and the Helsinki Declaration, i.e. the ICH Guideline for Good Clinical Practice (GCP) E6 of 1996, the CPMP/ICH/135/95 of September 1997 and its amendments [39-41]. The study is also in accordance with the German Federal Data Security Law (BDSG). All professionals participating at the study oblige themselves to adhere to the abovementioned declaration and law.

Study participation of patients is voluntary and can be cancelled at any time without provision of reasons and without negative consequences for their future medical care.

#### Patient informed consent

Previous to study participation patients receive written and spoken information about the content and extent of the planned study; for instance about potential benefits for their health and potential risks. In case of acceptance they sign the informed consent form. In case of study discontinuation all material will be destroyed or the patient will be asked if he/she accepts that existing material can be analysed in the study.

### Legal principles

#### Vote of the ethics committee

The study protocol was approved by the ethics committee of the University of Heidelberg previous to the start of the study in November 2006. Inclusion of patients/participants did not start unless there was a written and unrestricted positive vote of the ethics committee (approval number 303/2006). The study protocol was also approved by the ethics committee of the medical association of the state of Baden-Württemberg (approval number B-244-06-f). Both votes were received in October 2006. The study is registered (ISRCTN30822978).

#### Data security/disclosure of original documents

The patient names and all other confidential information fall under medical confidentiality rules and are treated according to German Federal Data Security Law (BDSG). The patient questionnaires and original CRFs are collected by the study nurse and mailed to the KKSL. These study related data and documents are stored on a protected central server of the KKSL. Only direct members of the internal study team can access the respective files.

Intermediate and final reports as well as the documentation of the Case management are stored in the office of the Department of General Practice and Health Services Research at the University Hospital Heidelberg.

The press copies of the clinical data and the documentation of the case management are stored in the centres for at least 10 years.

## Discussion

As the targeted patient group is mostly cared for and treated by GPs, a comprehensive primary care-based guideline implementation including somatic, psychosomatic and organisational aspects of the delivery of care (HICMAn) is a promising intervention applying proven strategies for optimal care of patients with CHF.

## Competing interests

The author(s) declare that they have no competing interests.

## Authors' contributions

FPK, TMT, AB, DS, WH and JS conceived and performed the study. FPK, TMT, JG and CM developed the intervention. All authors contributed to, read and approved the final manuscript.

## Pre-publication history

The pre-publication history for this paper can be accessed here:


